# Exploring pre-MRI imaging tests: patient survey reveals potential implications for healthcare efficiency in Israel

**DOI:** 10.1186/s13584-024-00593-0

**Published:** 2024-04-01

**Authors:** Arielle Kaim, Clara Singer, Lucia Bergovoy-Yellin, Osnat Luxenburg, Sharona Vaknin, Noga Boldor, Rachel Wilf-Miron, Vicki Myers

**Affiliations:** 1 The Gertner Institute for Epidemiology and Health Policy Research, Sheba Medical Center, Tel Hashomer, 5266202 Ramat-Gan, Israel; 2 Department of Emergency and Disaster Management, Faculty of Medicine, School of Public Health, Tel Aviv University, P.O. Box 39040, 6139001 Tel Aviv, Israel; 3 Medical Technology, Health Information and Research Directorate, Ministry of Health, Jerusalem, Israel; 4 Department of Health Promotion, Faculty of Medicine, School of Public Health, Tel Aviv University, P.O. Box 39040, 6139001 Tel Aviv, Israel

**Keywords:** Health policy, Radiology, Efficiency, Medical imaging

## Abstract

**Background:**

Medical imaging tests are vital in healthcare but can be costly, impacting national health expenditures. Magnetic resonance imaging (MRI) is a crucial diagnostic tool for assessing medical conditions. However, the rising demand for MRI scans has frequently strained available resources. This study aimed to estimate the prevalence of different imaging tests in individuals who eventually had an MRI, in the Israeli public health system.

**Methods:**

An online survey of patient experience of scheduling an MRI was conducted in January–February 2023, among 557 Israeli adults, representing all four health maintenance organizations (HMOs). All participants had undergone an MRI in the public health system within the past year.

**Results:**

Results showed that 60% of participants underwent other imaging tests before their MRI scan. Of those, computed tomography (CT) scans (43%), X-rays (39%), and ultrasounds (32%) were the most common additional imaging procedures. In addition, of the 60% of participants, 23% had undergone more than one prior imaging examination.

**Conclusions:**

These findings highlight the high prevalence of preliminary imaging tests prior to MRI, with many patients undergoing multiple tests for the same problem. The health system may need to evaluate whether current clinical guidelines defining the use of various imaging tests are cost-effective.

**Supplementary Information:**

The online version contains supplementary material available at 10.1186/s13584-024-00593-0.

## Introduction

Imaging tests play a vital role in diagnostic processes, treatment planning, and patient monitoring [1]. Among various imaging modalities, Magnetic Resonance Imaging (MRI) is a widely utilized and highly advanced method. However, its cost is relatively high because of expensive hardware, software, and the need for skilled professionals to operate and interpret the imaging results it [2]. Simultaneously, the demand for MRI is substantial due to its unique innovative features, non-ionizing safety compared to Computed Tomography (CT) scans, and its significance in diagnosis, including emerging clinical applications [3]. Additionally, factors like the aging population and increasing incidence of chronic diseases have contributed to the growing demand for MRI worldwide, including in Israel [4].This surge in demand, combined with a shortage of imaging personnel, has resulted in long waiting times and reduced patient satisfaction in Israel [3,5]. Despite this, individuals may choose to opt to pay for an MRI privately at a public institution.

As a result of lengthy waiting times, in 2016, the Israeli Ministry of Health initiated a national program to enhance the accessibility and availability of MRI examinations within the public healthcare system throughout the country [3]. This program involves a comprehensive intervention encompassing infrastructure improvements, workforce development, and financial incentives for the HMOs to increase volume of tests and shorten wait times (WTs). Initially, the program led to a reduction in MRI WT, but after a two-year period, there was a resurgence in WT. In 2021, the national average WT for an MRI examination was 53.3 days (7.6 weeks), while for CT the average was 14.8 days (2.1 weeks) according to the Israeli Ministry of Health in 2021.This challenge mirrors international trends, as evidenced by prolonged MRI wait times in countries with universal healthcare systems, including Canada [6] and the United Kingdom [7].As of March 2023, 25% of NHS England patients waited over 6 weeks for an MRI [6], and Canadian patients expected WTs of 4.9 weeks for ultrasounds, 5.4 weeks for CT scans, and 10.6 weeks for MRIs in 2022 [7].

The latest OECD report underscores a paradox in Israel's medical imaging capacity. Despite having fewer medical scanners per capita compared to other nations—with Israel near the bottom of the OECD rankings in terms of scanner availability—the country demonstrates a middle-range performance for the number of examinations per population [8].Other imaging modalities are available, each with benefits and limitations, including CT, ultrasound, or nuclear medicine techniques like positron emission tomography (PET). These methods offer both advantages, such as lower equipment costs, less complex infrastructure requirements, potentially faster scan times and greater availability compared to MRI [2], and drawbacks such as radiation exposure for some methods. The challenge for clinicians is to choose the most appropriate test, factoring in clinical indication, cost, availability, and radiation risk [9,10].

Healthcare systems strive to achieve a balance between cost-effectiveness and diagnostic efficacy by exploring and embracing alternative imaging techniques. It is essential to ensure that these alternative methods maintain a high level of diagnostic accuracy, meet patients' clinical needs effectively, and assure that the diagnostic pathway is efficient.

The aim of the current study was to estimate the percentage of individuals who received initial imaging tests prior to undergoing an MRI scan in Israel for the same clinical indication. The purpose of conducting such an investigation is to gain insights into the current practices and utilization of alternative imaging modalities in the diagnostic process, and whether MRIs are currently underutilized and should be more prominently positioned at the forefront of diagnostic strategies. The clinical imaging guidelines in Israel rely on the standards set by the European Society of Radiology (ESR) [11].

## Methods

### Study design

The study was conducted during the fourth week of January to the second week of February 2023. A sample of the Israeli population (N = 557) who underwent MRI in the past year completed an online survey of patients’ experience of scheduling an MRI.

Participants were recruited through an online internet panel company, Geocartography, which consists of over 25,000 members, representing all geographic and demographic sectors of the Israeli population (https://www.geokg.com/). A stratified sampling method was used, based on the relative patient membership size in each of the four HMOs in Israel, as well as geographic regions.

### Participants

The sample size was determined based on 5% of the population who undergo MRI annually in Israel (466,400 in 2021). To partake in the study, the participants had to confirm their willingness to voluntarily participate. Eligibility criteria were being aged 18 + and having had an MRI in the public system within the last year. A screening question was utilized where the participant was asked if he had an MRI reimbursed by the HMO (Tofes 17) which excludes those that received an MRI during hospitalization, however does included patients who received a scan at an independent scanning center, The data were collected anonymously, following approval of the Institutional Ethics Committee of Tel Aviv University (number 0005172 from 06–07–2022).

### The study tool

The current study used a questionnaire that aimed to explore patients’ experience with the process of scheduling an MRI scan, alongside demographic characteristics. In addition, questions related to waiting times and appointment scheduling for MRI in the public health system. Specifically, the focus of the current manuscript relates to two items which were assessed (1) Before undergoing an MRI scan, did you have another imaging test, due to the same medical problem? (2) Which imaging test did you have prior to MRI?: CT, X-Ray, Ultrasound, PET, endoscopy, mammography, previous MRI or other. The study-tool was developed and revised by several experts in the imaging field (researchers, health care policy makers, and a radiologist). The tool was then pilot tested and revised following recommendations for improved clarity. The study tool is attached in Appendix 1 in Additional file 1.

### Statistical analysis

Descriptive statistics were used to analyze the characteristics of the sample. Pearson chi-square analysis was computed for correlations between demographic characteristics and prior imaging modality. P-values lower than 0.05 were considered statistically significant. All statistical analyses were performed using SPSS software version 28.

## Results

Study population: Respondents were 57.5% female and 42.5% male; 92.2% Jewish and 7.7% Arab population. A third (33.2%) were aged 18–34; 44.5% aged 35–54 and 22.2% were aged 55 or over. 29% were from the Northern region, 50% from the Centre and Tel Aviv regions, 10% from the South and 10% from the Jerusalem region. The survey population was as representative as possible of the population of those who undergo MRI, though the Centre region was over-represented, and the South and Jerusalem were slightly under-represented; the Arab population were also under-represented (8% vs 21% of the population).

**Fig. 1:**
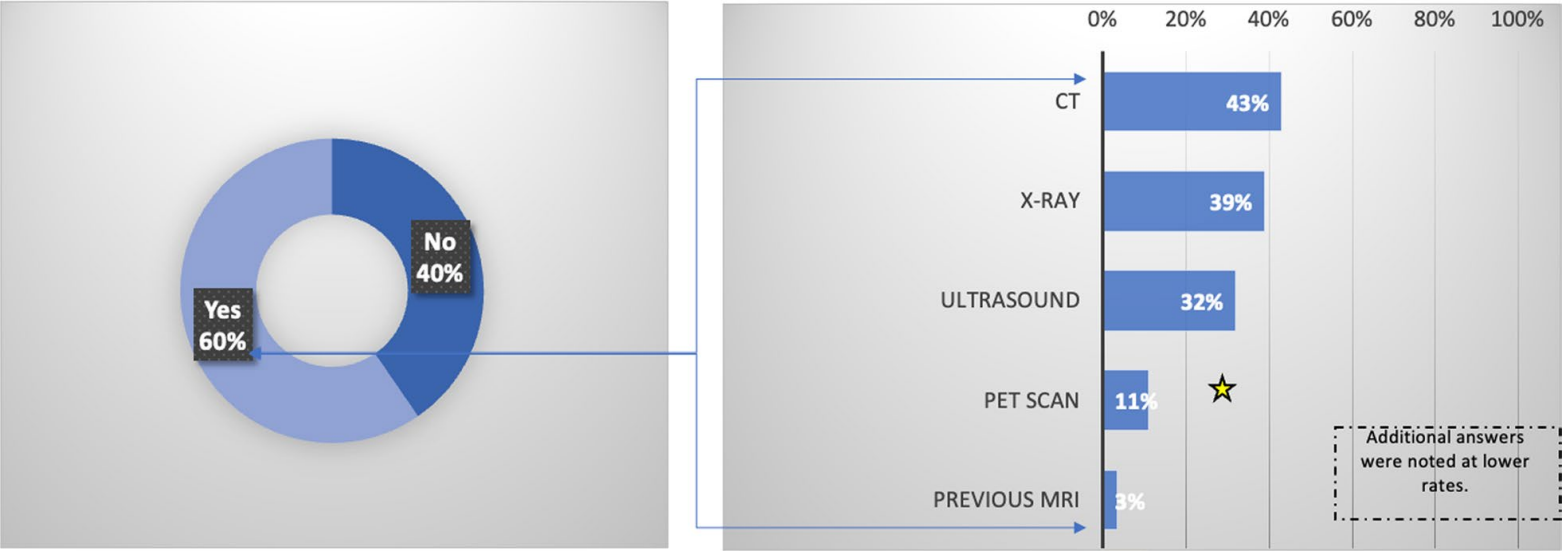
Percentage (%) of n = 557 that underwent a preliminary test prior to an MRI examination for the same problem, and the respective breakdown of imaging modality employed

Sixty percent of respondents underwent additional imaging procedures before their MRI scan (Fig. 1). Among those who received preliminary imaging tests, computed tomography (CT) scans were the most common, with 43.0% of participants undergoing this imaging modality. 23.1% of participants underwent more than 1 preliminary imaging modality test prior to the MRI scan among those who had prior imaging.

**Fig. 2:**
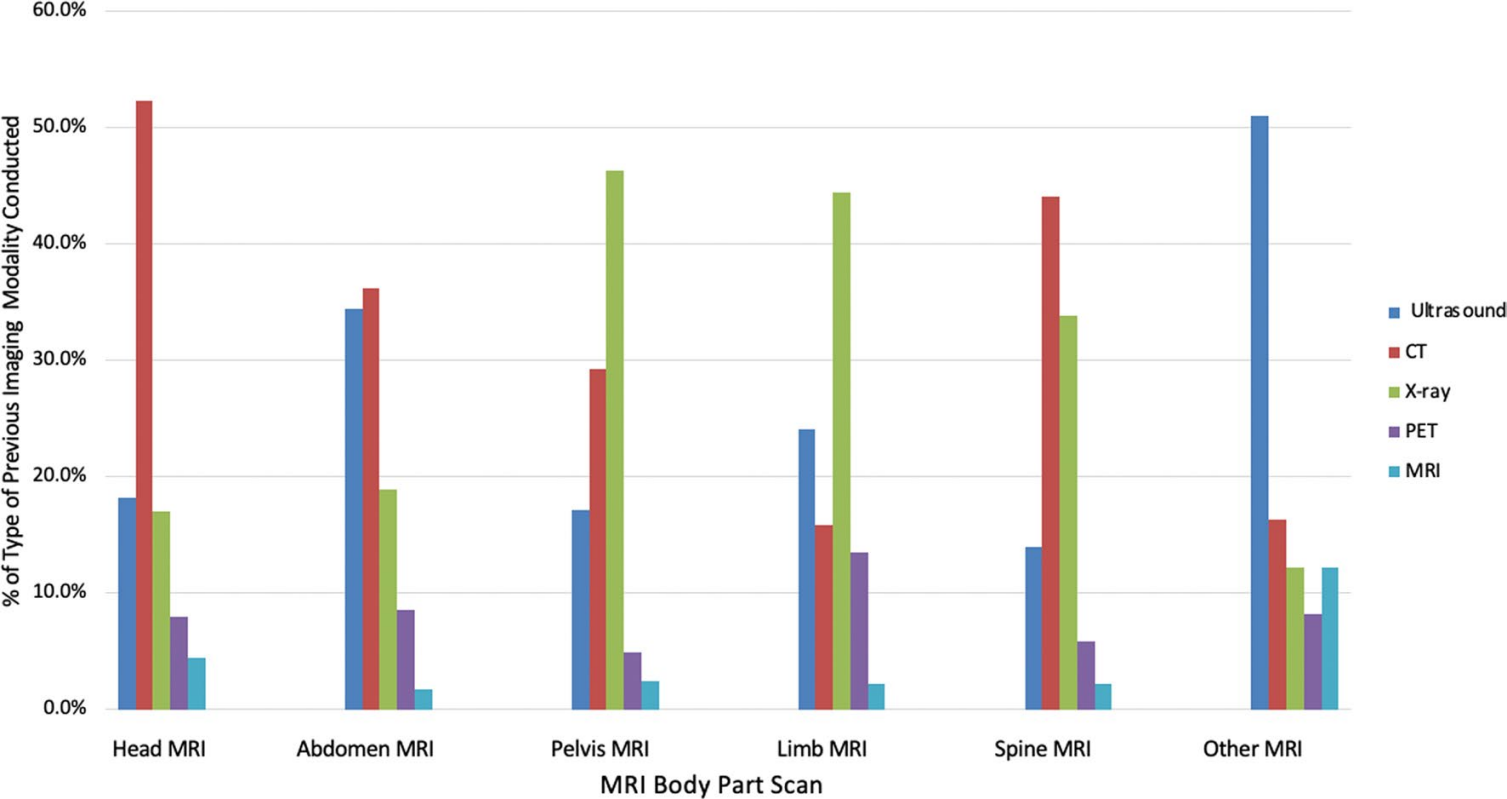
Percentage (%) of previous imaging modality (Ultrasound, CT, X-ray, PET, additional MRI) conducted, by MRI body part scanned. *Note*: Other MRI includes breast, heart, prostate, shoulders, neck, and thyroid

Furthermore, we analyzed the distribution of past imaging techniques performed on different body parts using MRI, as illustrated in Fig. 2. The results reveal that for individuals who underwent MRI scans of the head, abdomen, or spine, the prevailing previous imaging method employed was CT. In the case of MRI scans of the limbs or pelvis, x-ray emerged as the most frequently used modality, whereas ultrasound was found to be the predominant modality for other parts (including breast, heart, prostate, shoulders, neck, and thyroid) of the body examined.

Although a higher proportion of women underwent prior imaging examinations in various modalities, the difference was not significant (55% vs. 45%). The distribution of previous imaging modalities followed a similar pattern, with more women undergoing prior ultrasound (57.7% vs. 42.3%), CT (56.1% vs. 43.9%), PET (69.4% vs. 30.6%), and MRI (56.3% vs. 43.8%). However, for previous x-ray imaging, both men and women had relative equal participation. Significant differences were observed in relation to age and income. The highest rate of prior imaging was seen in the 35–54 year age group (48%) compared to 25% among 18–34 year olds and 28% in the 55 + group. Regarding income, a significant difference was found, with the highest proportion of prior imaging reported in the above average income group (36%) compared to 29% of those with below average income and 26.3% among those with average income. See Table 1.

**Table 1:** Demographic characteristics by previous imaging

	Prior Imaging (%) n = 334	*p* value	Prior US (%) n = 105	*p* value	Prior CT (%) n = 143	*p* value	Prior X-ray (%) n = 131	*p* value	Prior PET (%) n = 37	*p* value	Prior MRI (%) n = 16	*p* value
Sex												
Male	45	0.161	42.3	0.964	43.9	0.706	50	0.141	30.6	0.133	43.8	0.919
Female	55		57.7		56.1		50		69.4		56.3	
Age												
18–34	24.6	< 0.001	35.0	0.101	35.5	0.130	35.2	0.085	34.0	0.102	33.1	0.512
35–54	47.9		44.1		42.5		44.4		44.6		44.9	
55–65 +	28.4		20.8		22.0		20.4		21.3		22.0	
Income												
Below average	29	0.008	39.6	0.518	24.5	0.024	31.3	0.009	29.7	0.914	25.1	0.422
Average	26.3		38.8		28.7		16.8		29.7		18.8	
Above average	36.3		29.3		36.4		41.2		32.4		43.8	

Statistical significance assessed with chi- square test. *p* Values lower than 0.05 considered to be statistically significant

## Discussion

The high prevalence of prior imaging tests before MRI scans, as indicated by the survey findings, highlights the need for a comprehensive evaluation of current diagnostic protocols and pathways [12]. The findings raise concerns about potential inefficiencies and redundant examinations within the Israeli healthcare system, as has been assessed in other countries [13].Various imaging modalities are often used to save time, or costs as a first alternative to MRI, where wait times are long. What the current study shows is that many patients end up doing several tests and then also having an MRI. The question for clinicians is whether current protocols provide the most efficient and cost-effective pathway to diagnosis? By identifying in which cases patients are most likely to end up getting an MRI, it might be possible to reduce additional testing usually carried out prior to the MRI. While these preliminary tests serve the purpose of screening and evaluating patients, it is important to assess whether they are truly necessary, considering that an MRI examination is ultimately required. Redundant examinations, where multiple imaging tests are conducted preceding an MRI, can lead to delayed diagnosis, decreased patient satisfaction, increased anxiety, and increased healthcare costs, ultimately longer waiting times, and unnecessary radiation exposure for patients [14]. Previous work has similarly pointed to analogous conclusions such as Ip et al., (2012), who explored repeat (0–90 days following) abdominal imaging examinations for the same clinical problem in the United States [15]. The research team suggests that it may be reasonable to consider bypassing x-ray or ultrasound in favor of directly using CT or MRI to alleviate the repeat testing and diagnosis delays. By evaluating the cost-effectiveness and clinical efficacy of preliminary imaging tests, this would allow healthcare providers to make informed decisions regarding the utilization of different imaging modalities, optimizing resource allocation and potentially reduce overall healthcare expenditure.

In addition, to facilitate consistent and standardized practices, imaging referral guidelines should be re-evaluated and standardized between service providers (HMOs) for national-level clinical guidelines to ensure optimal use [16,17]. These guidelines should define the appropriate use of various imaging tests before an MRI, based on evidence-based research and expert consensus, in addition to considering factors such as the suspected medical conditions, the potential benefits and risks of each imaging modality, and likelihood of requiring follow-up imaging examination. Such guidelines would promote uniformity in clinical decision-making, reducing unwarranted variations in practice and ensuring that patients receive consistent and high-quality care regardless of the healthcare setting [18].

The findings from this study also shed light on the patterns of prior imaging examinations with regard to different demographic characteristics of those undergoing prior imaging. While a greater percentage of women underwent imaging examinations across the various modalities, the lack of significance in the relationship suggests that gender may not be a determining factor in performing prior imaging. The observed variations across age groups and income levels highlight the influence of these factors on the likelihood of undergoing prior imaging. Specifically, the mid-adult age group had the highest percentage of prior imaging, while the young adult and elder age groups showed lower rates. Moreover, individuals with above average income had a higher propensity for prior imaging compared to those with average or below average income. Previous work has indicated that demographic characteristics have played a role in appropriate imaging medical referral (e.g., women were more likely to have an imaging test referral classified as appropriate)^19^.These findings emphasize the importance of considering demographic factors when evaluating imaging utilization and healthcare disparities, which can inform targeted interventions to ensure equitable access and appropriate utilization of imaging services. Further examination is warranted to explore the underlying reasons behind these observed differences and their impact on patient outcomes.

While this study provides preliminary insights into the proportion of individuals undergoing preliminary imaging tests before an MRI scan, it is important to acknowledge substantial limitations of the study design, including limited generalizability given that the study sample consisted of 557 Israeli adults who received an MRI within the public health system. The sample was not fully representative of the general population, with under-representation of the Arab population and of the Southern region. The sample was taken from an Internet panel, thus would not represent people with low digital literacy. Furthermore, the data collected in this study relied on self-reported information through an online survey. There is a potential for recall bias or misinterpretation of the questions by participants, leading to inaccuracies in the reported prevalence of preliminary imaging tests. Lastly, the survey focused on the proportion of individuals who underwent prior imaging tests before an MRI scan, but it did not capture detailed clinical information about the specific medical conditions or indications for the imaging tests on a case-by-case basis, nor did it compare the results of the initial and subsequent tests to see if they matched or differed. Without this clinical context, it is challenging to fully evaluate the appropriateness or necessity of the preliminary tests and thus interpretation of the preliminary findings of this study must be taken with caution. As this is a preliminary, exploratory study, a crucial aspect of future research would be to ascertain the clinical indications that dictate the choice of imaging—be it MRI as a primary diagnostic tool or as a secondary option for confirmation and staging purposes. This study underlines also the potential necessity of multiple imaging tests to ensure accurate diagnosis and patient care. Further investigation should aim to clarify these protocols and optimize patient work-up strategies. Addressing these limitations in future research can further enhance our understanding of the appropriateness, effectiveness, and cost implications of preliminary imaging tests before MRI scans, leading to more evidence-based clinical guidelines and optimized resource allocation.

## Conclusions

Given the substantial occurrence of multiple tests preceding necessary MRI procedures, which not only prolongs the diagnostic journey but also imposes psychological stress on patients, a critical evaluation of current practices is essential. The implications of such practices extend to healthcare quality and financial efficiency. Future research must rigorously examine and refine testing protocols, ensuring they are clinically justified and sensitive to patient needs. This will enhance the patient experience by alleviating distress associated with prolonged diagnostic timelines, and possibly improve overall patient healthcare outcomes. Moreover, this may lead to optimized resource utilization, and a reduction in unnecessary expenses.

## Supplementary Information

**Additional file 1. Appendix 1.** Study Tool.

## Data Availability

The datasets during and/or analysed during the current study available from the corresponding author on reasonable request.

## Abbreviations

MRI: Magnetic resonance imaging
CT: Computed tomography
PET: Positron emission tomography
WTs: Wait times
HMOs: Health maintenance organizations
