# Supplementary material for: Exploring pre-MRI imaging tests: patient survey reveals potential implications for healthcare efficiency in Israel
Source: Isr J Health Policy Res. 2024 Apr 1;13:7. doi: 10.1186/s13584-024-00593-0 (PMC10983758; doi:10.1186/s13584-024-00593-0)
Supplement: Supplementary file 1 — Additional file 1. Appendix 1. Study Tool. [file 13584_2024_593_MOESM1_ESM.docx]

**שאלון MRI***הגדרת להכללה במחקר: MRI במסגרת שירותי בריאות ציבוריים בלבד, עבורו ניתנה התחייבות (טופס 17) מהקופה – ללא ביצוע במסגרת פרטית, ללא חיילים שבצעו במסגרת צבאית*

**אנו מודים לך על הנכונות להשתתף בסקר. השאלות מתייחסות לתהליך של קביעת התור לבדיקת MRI במערכת הרפואה הציבורית, דעתך חשובה לנו. תשובותיך יישארו חסויות ולא יועברו לגוף שלישי.**

**דמוגרפיה**

**מין** 1. זכר 2. נקבה 3. אחר

**גיל/שנת לידה** ______

**ישוב מגורים** _____

**קופת חולים בה את/ה מבוטח/ת**: 1. כללית 2. מכבי 3. מאוחדת 4. לאומית 5. אחר

**דת - האם את/ה:**

1. יהודי
2. מוסלמי
3. נוצרי
4. דרוזי
5. אחר
6. מסרב לענות

**כיצד תגדיר/י את רמת הדתיות שלך:**

1. חילוני
2. מסורתי
3. דתי
4. חרדי
5. אחר

**השכלה:**

1. סיום יסודי/חטיבה
2. סיום תיכון
3. על-תיכונית/ אקדמית (אוניברסיטה, מכללה)
4. אחר __________

**הכנסה –**

ההכנסה הנטו הממוצעת למשק בית בישראל בשנת 2019 הייתה 16.600 ₪. האם סך ההכנסה המשפחתית שלכם:

1. מתחת לממוצע
2. ממוצע
3. מעל הממוצע
4. מסרב לענות

**שאלות על קביעת התור לבדיקת MRI**

1. **לאיזה איבר בגוף בוצעה בדיקת ה MRI** (ניתן לסמן יותר מתשובה אחת):
2. ראש
3. בטן
4. אגן
5. רגליים / ידיים
6. עמוד שדרה
7. אחר ________________

2**. האם לפני ביצוע בדיקת MRI ביצעת בדיקת דימות נוספת לאותה בעיה רפואית:**

א. כן

ב. לא

3. **אם כן, באמצעות איזה מהמכשירים הבאים בוצעה הבדיקה:**

א. אולטרסאונד

ב. CT

ג. צילום רנטגן

ד. מיפוי

ה. אחר _______________

1. **חודש/שנה בו בוצעה בדיקת ה- MRI** ____/___
2. **זמן הבדיקה:**
3. בוקר
4. צהרים
5. ערב
6. לילה
7. **איפה בוצעה הבדיקה**:
8. בית חולים
9. מכון
10. ניידת
11. **שם המכון או בית חולים**___________
12. **שם הישוב בו בוצעה הבדיקה:** ____________
13. **מה הייתה סיבת הבדיקה**:
14. בירור ראשוני
15. בדיקת מעקב (אחת מסדרת בדיקות לאותה הבעיה הרפואית )

10. **כמה זמן עבר** מרגע שקיבלת את ההפניה הכתובה ל- MRI, ועד הרגע שפנית לבית חולים/מכון בו בוצעה בדיקת ה-MRI לצורך קביעת תור?

1. עד שבוע
2. שבוע עד שבועיים
3. שבועיים עד חודש
4. חודש עד חודשיים
5. מעל חודשיים
6. לא זוכר/ת

11. **כמה זמן המתנת מרגע שניסית לקבוע תור ועד ליום ביצוע הבדיקה ?**

1. עד שבועיים
2. שבועיים עד חודש
3. חודש עד חודשיים
4. חודשיים עד 4 חודשים
5. 4-6 חודשיים
6. מעל 6 חודשים
7. לא זוכר/ת

12. **כמה זמן חלף מרגע ההמלצה של הרופא בקופה, עד לקבלת ההתחייבות מהקופה (טופס 17) ?**

א. עד שבוע

ב. שבוע עד שבועיים

ג. שבועיים עד חודש

ד. מעל חודש

13**. האם הסכמת לקבל את תור הראשון (המוקדם ביותר) שהוצע לך לבדיקה?**

1. כן
2. לא

14. **האם ניסית להקדים את התור שהוצע לך ?**

1. כן
2. לא

15. **האם התור הוקדם או נדחה ?**

1. הוקדם על ידי בית חולים/מכון MRI
2. נדחה על ידי בית חולים/ מכון MRI
3. נדחה על ידי
4. נותר ללא שינוי

16**. מה הייתה הסיבה שביצעת את הבדיקה באותו בי"ח / מכון MRI ?**

1. מיקום נוח
2. התור הכי מהר
3. הרופא /קופה הפנו אותי /המליצו לי
4. לא ידעתי שיש אופציה אחרת
5. אחר

17**. עד כמה התקשית בתהליך קביעת התור ל-MRI?**

1. לא התקשיתי בכלל
2. לא כל כך התקשיתי
3. התקשיתי
4. התקשיתי מאד

18**. הזמן הכולל שהמתנתי לבדיקה (מהפנייה ועד ביצוע בפועל) היה: (נא לדרג על סולם בין 1-5)**

סביר __________________ לא סביר

1 2 3 4 5

19. **כמה זמן אחרי הבדיקה קיבלת את הפענוח הרפואי ?**

א. עד שבוע

ב. שבוע עד שבועיים

ג. שבועיים עד חודש

ד. מעל חודש

ה. לא זוכר

ו. לא קיבלתי

20**. עד כמה הזמן עד לקבלת הפענוח היה סביר בעיניך? (נא לדרג על סולם בין 1-5)**

סביר __________________לא סביר

1 2 3 4 5

21**. כמה קל היה כל אחד מהשלבים הבאים בתהליך? נא לסמן בטבלה**

|  | מאד קל | קל | מעט קשה | קשה | קשה מאוד |
| --- | --- | --- | --- | --- | --- |
| לקבל אישור והתחייבות מהקופה (טופס 17) |  |  |  |  |  |
| לקבוע תור |  |  |  |  |  |
| להבין לאן לפנות (כדי לקבוע תור) |  |  |  |  |  |
| לקבל פענוח |  |  |  |  |  |
| אחר _________ |  |  |  |  |  |
